# Metabolic syndrome and osteoporotic fracture: a population-based study in China

**DOI:** 10.1186/s12902-016-0106-x

**Published:** 2016-05-27

**Authors:** Li Qin, Zhen Yang, Weiwei Zhang, Hongxia Gu, Xiaoyong Li, Lingfei Zhu, Shuai Lu, Yin Xing, Hongmei Zhang, Yixin Niu, Guang Ning, Qing Su

**Affiliations:** Department of Endocrinology, Xinhua hospital Chongming branch, School of medicine, Shanghai Jiaotong University, 25 Nanmen Road, Shanghai, China; Department of Endocrinology, Xinhua hospital, School of medicine, Shanghai Jiaotong University, Shanghai, China; Department of Endocrinology, Ruijin hospital, School of medicine, Shanghai Jiaotong University, Shanghai, China

**Keywords:** Metabolic syndrome, Osteoporotic fractures, Central obesity

## Abstract

**Background:**

Associations between metabolic syndrome (MetS) and osteoporotic fracture have been reported. However, the epidemiological studies are not conclusive. The objective of the study was to determine whether metabolic syndrome associates with osteoporotic fracture.

**Methods:**

This was a cross-sectional study of 9930 Chinese adults aged 40 year or older in the Chongming District, Shanghai, China. A questionnaire, anthropometric measurements and laboratory tests were conducted. MetS was defined according to the updated National Cholesterol Education Program Adult Treatment Panel III criteria for Asian-Americans. A history of fractures was collected with an interviewer-assisted questionnaire. Osteoporotic fractures were defined as fractures that occurred due to low-trauma in 2 years prior to the study.

**Results:**

Among women, the prevalence of osteoporotic fractures was significantly higher in those with MetS (3.5 vs. 2.6 %, *P* =0.028). However, the difference was not found in men (2.6 vs. 2.4 %, *P* =0.737). The presence of Mets was significantly associated with increased odds of osteoporotic fracture among women (odds ratio 1.22; 95 % confidence interval 1.12–1.54; *P* = 0.039) after controlling for potential confounders. The significant associations were not detected in men.

**Conclusions:**

The presence of MetS was significantly associated with a recent history of osteoporotic fracture in middle-aged and elderly Chinese women.

**Electronic supplementary material:**

The online version of this article (doi:10.1186/s12902-016-0106-x) contains supplementary material, which is available to authorized users.

## What’s new?

- ✓In this study we found the presence of MetS was significantly associated with a recent history of osteoporotic fracture in a large sample of middle-aged and elderly Chinese women.
- ✓We found central obesity seem to have the strongest associations with prevalence of osteoporotic facture in women.
- ✓Our study indicated bone metabolism seems to be more markedly affected in women than in men with metabolic syndrome.

## Background

With the rapid economic growth and the ageing of its population in China, osteoporosis has become one of the most prevalent and debilitating chronic diseases. It has often been called “the silent epidemic” because bone loss is a slow asymptomatic process. Only when fractures occur does the disease become symptomatic. Recently, osteoporotic fracture has become an increasingly important problem in elderly populations and place a progressively heavy burden on health services [1, 2]. Nutritional, lifestyle, hormonal, metabolic, and genetic factors contribute to the occurrence of osteoporotic fracture [2].

Metabolic syndrome (MetS) is a cluster of cardiovascular risk factors has been defined as central obesity, hypertension, impaired glucose tolerance, and dyslipidemia (specifically hypertriglyceridemia, along with low levels of high-density lipoprotein cholesterol [HDL-c]). It is a public health problem because about 30–50 % of the middle-aged and elderly people has MetS in China [3, 4]. MetS is associated with a higher risk of cardiovascular morbidity and mortality and a higher risk of onset of type 2 diabetes. Although the relationship between cardiovascular disease and osteoporosis has been widely studied, the particular association between metabolic syndrome, a strong risk factor for cardiovascular disease, and osteoporotic fractures has not been so extensively addressed and present several studies results were inconsistent [5–15]. Epidemiological studies on fracture incidence or prevalence are therefore needed in order to show the real pattern of fractures in MetS. In addition, because metabolic syndrome consists of several single components that could have their own independent relationship with osteoporosis, the possible association of each of them with the osteoporotic fracture is also worth considering.

Thus, the present study intended to evaluate the association between MetS and osteoporotic fracture in a population-based survey of middle-aged and elderly Chinese people in Shanghai, China. We also examined the association of each of the individual components of metabolic syndrome with osteoporotic fracture.

## Methods

### Study population and design

In 2011 China a national survey of Risk Evaluation of cAncers in Chinese diabeTic Individuals: a lONgitudinal (REACTION) study, which was conducted among 259,657 adults, aged 40 years and older in 25 communities across mainland China, from 2011 to 2012 [16]. The data presented in this article are based on the baseline survey of subsamples from Shanghai in eastern China. All studied individuals came from the Chongming District in Shanghai, China [17–19]. Written consent was obtained from all the participants. The protocol was approved by the Ethics Committee of Xinhua Hospital Affiliated to Shanghai Jiaotong University School of Medicine.

### Data collection

A standardized questionnaire was used by trained physicians to collect information such as age, sex, medications [17–19]. The methods of biochemical measurements refer as previous studies [17–19]. Physical activity at leisure time was estimated using the short form of the International Physical Activity Questionnaire by adding questions on frequency and duration of moderate and vigorous activities and walking [20]. For evaluation of total physical activity, separate metabolic equivalent hours per week (MET-h/wk) were calculated.

Anthropometric measurements were performed by the trained personnel using a standardized protocol [17–19]. BMI was calculated as body weight in kilograms divided by height squared in meters. The abbreviated Modification of Diet in Renal Disease formula recalibrated for Chinese [21].

### Definition of MetS

The MetS was defined based upon the updated NCEP-ATPIII for Asian Americans as presenting 3 or more of the following components: 1) waist cirumference ≥90 cm for men or ≥80 cm for women; 2) triglycerides ≥1.7 mmol/l; 3) HDL cholesterol <1.03 mmol/l for men or <1.30 mmol/l for women; 4) blood pressure ≥130/85 mm Hg or current use of anti-hypertensive medications; and 5) fasting glucose ≥5.6 mmol/l or previously diagnosed type 2 diabetes or on oral antidiabetic agents or insulin [22].

### Assessment of fractures

With an interviewer-assisted questionnaire, we collected information about the history of fractures. Fracture sites and the age of the participants when they had fractures were recorded. For the current study, osteoporotic fractures were defined as fractures that occurred due to low trauma in the 2 yr before the study [23]. All fractures that were considered to be nonosteoporotic (i.e. fractures due to cancer or an accident, such as a motor vehicle accident, and all fractures of the fingers, faces, skull, and toes) were excluded [24].

### Statistical analysis

The statistical analysis was conducted using SPSS (version 13.0, SPSS Inc., Chicago, Illinois). Because sex is an important confounding factor of osteoporotic fractures, we performed analyses separately in men and women [25]. The following skewed variables were normalized by logarithmic transformation before analyses in which they were treated as continuous variables: TG, eGFR and MET-h/wk. All continuous variables were presented as means ± SD or medians (inter-quartile ranges), and all categorical variables were presented as proportions. The t test and Chi-square test were used to examine differences in continuous and categorical variables, respectively. To investigate the associations between metabolic syndrome and osteoporotic fracture, the unadjusted and multivariate adjusted logistic regression analyses were used to assess odds ratios (OR) and corresponding 95 % confidence intervals (CI). The statistical tests were two sided, and a *P* < 0.05 was considered statistically significant.

## Results

Among the 9930 participants, the mean (±SD) age was 56.2 ± 7.8 yr, 32.5 % were men. The overall prevalence of osteoporotic fracture was 2.9 % (2.5 % in men and 3.1 % in women). The skeletal sites of the most common fractures were wrist in men (22.2 % of all fractures, *n* = 18) and ankle in women (20.2 % of all fractures, *n* = 42).

The clinical and biochemical characteristics stratified by metabolic syndrome status are shown for men and women in (Additional file 1: Table S1A and S1B). In men, participants with metabolic syndrome were older, more likely to be obese, and had significantly higher levels of TG, LDL-c, fasting plasma glucose (FPG), postprandial 2-h plasma glucose (PPG), HbA1c, waist circumference (WC), Hip circumference (HC), waist to hip ratio (WHR) and alcohol consumption habits and lower levels of HDL-c and eGFR (all *p* < 0.01). There was no significant difference between the two groups for TC, physical activity (MET-h/wk), and smoking. Similarly, in women, higher levels of age, BMI, WC, HC, WHR, TG, LDL-c, FPG, PPG, and alcohol consumption habits and lower levels of HDL-c were found in those with metabolic syndrome (all *p* < 0.05). Women with metabolic syndrome were less likely to be postmenopausal. However, the levels of serum TC in women with metabolic syndrome were higher in comparison with those without metabolic syndrome (*p* < 0.0001).

We further investigate the prevalence of osteoporotic fractures according to metabolic syndrome status for men and women. Among women, the prevalence of osteoporotic fractures was significantly higher in those with metabolic syndrome (3.5 vs. 2.6 %, *P* =0.028). However, the difference was not found in men (2.6 vs. 2.4 %, *P* =0.737).

As shown in Table 1, the presence of metabolic syndrome was significantly associated with increased odds of osteoporotic fracture among women (OR 1.38; 95 % CI 1.04–1.85; *P* =0.028). Further adjustment for age, smoking and alcohol consumption habits (yes/no), physical activity (MET-h/wk), eGFR, BMI, waist circumference and hip circumference, TG, TC, HDL-c, LDL-c, diabetes status (yes/no), FPG, HbA1c, menopause status (yes/no) and hormone replacement (yes/no) treatment did not materially change the association (OR 1.22; 95 % CI 1.12–1.54; *P* = 0.039). However, the significant associations were not detected in men.

**Table 1:** Association between metabolic syndrome and a recent history of osteoporotic fracture

	Men		Women	
	OR (95 % CI)	*P* value	OR (95 % CI)	*P* value
unadjusted	1.08 (0.70–1.68)	0.737	1.38 (1.04–1.85)	0.028
Model 1	0.96 (0.73–1.57)	0.596	1.25 (1.02–1.69)	0.031
Model 2	0.87 (0.68–1.44)	0.264	1.20 (1.02–1.57)	0.034
Model 3	1.00 (0.74–1.36)	0.986	1.23 (1.09–1.45)	0.038
Model 4	1.00 (0.73–1.36)	0.991	1.21 (1.11–1.42)	0.038
Model 5	1.01 (0.75–1.61)	0.784	1.18 (1.13–1.39)	0.039
Model 6			1.20 (1.11–1.53)	0.040

We defined participants without osteoporotic fractures as 0 and those with osteoporotic fractures as 1. Model 1 was adjusted for age, smoking and alcohol consumption habits (yes/no), physical activity (MET-h/wk), and eGFR. Model 2 was further adjusted for BMI, waist circumference and hip circumference based on model 1. Model 3 was further adjusted for serum TG, TC, HDL-c, and LDL-c based on model 2. Model 4 was further adjusted for diabetes status (yes/no), FPG and HbA1c based on model 3. Model5 was further adjusted for blood pressure on model 4. Model 6 was further adjusted for menopause status (yes/no) and hormone replacement (yes/no) treatment for women based on model 5

Tables 2 and 3 shows that Ors for the fracture risk calculation according to increasing numbers of the metabolic syndrome by ATPIII and IDF criteria. The fracture risk significantly increased according to increasing numbers of the metabolic syndrome by ATPIII and IDF criteria in women, but not in men.

**Table 2:** The fracture risk calculation according to increasing numbers of the metabolic syndrome (ATPIIIcriteria)

	ORs (95 % CI)						
Numbers of components of MetS	0	1	2	3	4	5	*P* value for trend
Men	1	0.98 (0.71–1.31)	0.96 (0.67–1.29)	1.02 (0.74–1.37)	1.02 (0.76–1.40)	1.11 (0.95–1.44)	0.127
Women	1	0.97 (0.69–1.32)	1.01 (0.78–1.41)	1.26 (1.02–1.67)	1.39 (1.09–1.74)	1.54 (1.23–2.17)	0.005

We defined participants without osteoporotic fractures as 0 and those with osteoporotic fractures as 1. Adjusted for age, smoking, alcohol consumption habits (yes/no), physical activity (MET-h/wk), eGFR, BMI, menopause status (yes/no) and hormone replacement (yes/no) treatment for women

**Table 3:** The fracture risk calculation according to increasing numbers of the metabolic syndrome (IDF criteria)

	ORs (95 % CI)						
Numbers of components of MetS	0	1	2	3	4	5	*P* value for trend
Men	1	0.99 (0.72–1.32)	0.97 (0.69–1.30)	1.01 (0.75–1.36)	1.02 (0.75–1.39)	1.10 (0.94–1.43)	0.132
Women	1	0.98 (0.70–1.31)	1.02 (0.79–1.42)	1.28 (1.04–1.71)	1.42 (1.11–1.82)	1.58 (1.25–2.24)	0.002

We defined participants without osteoporotic fractures as 0 and those with osteoporotic fractures as 1. Adjusted for age, smoking, alcohol consumption habits (yes/no), physical activity (MET-h/wk), eGFR, BMI, menopause status (yes/no) and hormone replacement (yes/no) treatment for women

Table 4 shows the ORs for individual components of metabolic syndrome by NCEP-ATPIII for Asian associated with osteoporotic fractures. Of the five MetS components, hypertension and central obesity seem to have the positive associations with risk of osteoporotic fractures in women.

**Table 4:** Odds ratios and 95 % confidence interval (CI) of osteoporotic fracture for individual components of MetS in male and female subjects

	Men		Women	
	OR (95 % CI)^a^	*P* value	OR (95 % CI)^a^	*P* value
Individual components of MetS				
High blood pressure	1.30 (1.01–1.68)	0.045	1.18 (1.05–1.47)	0.034
Hyperglycemia	0.94 (0.68–1.28)	0.614	1.03 (0.78–1.33)	0.807
Hypertriglyceridemia	0.97 (0.70–1.33)	0.842	0.91 (0.67–1.24)	0.556
Low HDL cholesterol	1.03 (0.72–1.48)	0.885	1.12 (0.79–1.59)	0.532
Central obesity	1.13 (0.78–1.65)	0.516	1.71 (1.05–2.67)	0.023

^a^Adjusted for age, smoking, alcohol consumption habits (yes/no), physical activity (MET-h/wk), eGFR, BMI, waist circumference, hip circumference, TG, TC, HDL-c, LDL-c, diabetes status (yes/no), FPG, HbA1c, menopause status (yes/no) and hormone replacement (yes/no) treatment for women

## Discussion

In this study, we found that the presence of MetS was significantly associated with a recent history of osteoporotic facture in women aged 40 yr or older. Moreover, this association was independent of other potential traditional risk factors.

Metabolic syndrome (MetS) is a cluster of cardiovascular risk factors which have been grouped under this name because they coexist much more often than would be expected by chance. Whether it is a real clinical entity or not is the subject of much controversy, but in any case, apart from its cardiovascular consequences, a possible relationship with bone mass has been entertained [26–28]. Furthermore, some common mechanisms for both disorders have been proposed. For instance, low vitamin D levels are considered predisposing factors for osteoporosis, and have also been suggested to facilitate the development of MetS [29–32]. In addition, few studies have presented increased prevalence of osteoporosis fractures in subjects with MetS [11, 12]. In our study, after adjusted for potential cofounders, a positive association between MerS and a recent history of osteoporotic fracture was detected.

Our study indicated bone metabolism seems to be more markedly affected in women than in men with metabolic syndrome. The differences between men and women in bone structure and strength, body fat deposition, sex hormone levels, and risk of falls could be potential explanations for the inconsistency of associations between osteoporotic fracture and MetS by genders [32]. Potential sex-specific associations of MetS with osteoporotic fracture need to be elucidated further. Our findings lend support to the postulation that the presence of metabolic syndrome in middle-aged and elderly Chinese women may confer a higher osteoporotic fracture risk. These findings suggest an awareness of the need to screen women with MetS for the presence of osteoporosis in fracture prevention. Moreover, the potential contribution of metabolic syndrome to development of osteoporosis warrants further study. Furthermore, in order to investigate whether other definition of the MetS also confirm the finding, we have re-analyzed the results by definition of the MetS (IDF 2005), similarly, we also found that the presence of metabolic syndrome was significantly associated with increased odds of osteoporotic fracture among women. Simultaneously, we found he prevalence of fracture significantly increased for those with one to five components after adjustment for age, gender (P *trends* < 0.05).

To study the possible independent relationship between the five components of metabolic syndrome and osteoporotic facture, a logistic regression analysis was performed. Hypertension and central obesity seem to be the positive associations with risk of osteoporotic fractures in women. Consistent with our results, previous studies have found higher blood pressure in elderly women was associated with increased bone loss at the femoral neck [33]. Moreover, low fracture risk was observed among those using treatment for hypertension although it was not significant [34]. Recently, a large-scale case–control study have also demonstrated that hypertension seem to be the major cardiovascular risk factors for fractures [35]. Certainly, as most fractures occur in those elderly people and blood pressure increases with age, an increased risk of fractures for increasing blood pressure should be expected. Therefore, most available data point toward a positive association between both parameters, even if the pathogenetic mechanisms involved remain unclear.

We found central obesity seem to have the strongest associations with prevalence of osteoporotic facture in women. Although the pathophysiological mechanisms linking central obesity to osteoporotic fracture have not been well established, it is plausible to consider excessive accumulation of visceral fat could result in a higher secretion of proinflammatory cytokines, with a detrimental effect on bone; in fact, a negative relation between fat mass and bone mass for a given body weight has been reported [36–38].

From few bone histology studies in humans and experimental studies there is evidence that a decreased bone formation is one major mechanism leading to reduced bone mass in diabetics. Few studies address the role of diabetes and osteroporosis [39, 40]. However, we fail to replicate this results in our study. There is a need for further longitudinal studies, including the incidence and risk factors for osteoporotic fractures.

Several limitations of our study have to be addressed. First, due to the cross-sectional nature of the current study, admittedly, we could not determine whether MetS plays a causal role in the pathogenesis of osteoporotic fracture. Further prospective population-based studies are needed to clarify their precise interrelationship. Second, because the information of osteoporotic fracture is based on self-reports and no measurements of bone mineral density by dual-energy x-ray absorptiometry or other radiological methods were made, we could not identify asymptomatic osteoporotic fractures. Finally, although a wide spectrum of covariates were included in the adjustment, some residual or undetected confounding factors could not be ruled out, such as dietary calcium intake or serum 25-hydroxyvitamin D3 levels, which are other factors affecting osteoporosis [41].

## Conclusions

We have found that the presence of MetS was significantly associated with a recent history of osteoporotic fracture in middle-aged and elderly Chinese women. Further longitudinal studies are expected to determine the role of MetS in the development of osteoporotic fracture.

## Abbreviations

MetS, metabolic syndrome; HOMA-IR, Homeostasis model assessment of insulin resistance; BMI, body mass index; TG, triglycerides; TC, total cholesterol; LDL-c, low-density lipoprotein cholesterol; HDL-c, high-density lipoprotein cholesterol; eGFR, estimated glomerular filtration rate; ORs, odds ratios.

## Additional file

Additional file 1:
**Table S1A.** Characteristics of Men and Women Stratified by Metabolic Syndrome Status (ATPIII criteria). **Table S1B.** Characteristics of Men and Women Stratified by Metabolic Syndrome Status (IDF criteria). (DOC 103 kb)
