# Supplementary material for: Metabolic syndrome and osteoporotic fracture: a population-based study in China
Source: BMC Endocr Disord. 2016 May 27;16:27. doi: 10.1186/s12902-016-0106-x (PMC4882795; doi:10.1186/s12902-016-0106-x)
Supplement: Additional file 1: — Table S1A. Characteristics of Men and Women Stratified by Metabolic Syndrome Status (ATPIII criteria). Table S1B. Characteristics of Men and Women Stratified by Metabolic Syndrome Status (IDF criteria). (DOC 103 kb) [file 12902_2016_106_MOESM1_ESM.doc]

Supplement Table S1A. Characteristics of Men and Women Stratified by Metabolic Syndrome Status (ATPⅢ criteria)

|  | Men (n=3229) | | |  | women (n=6701) | | |
| --- | --- | --- | --- | --- | --- | --- | --- |
|  | MetS (-) | MetS (+) | P value |  | MetS (-) | MetS (+) | P value |
| n (%) | 1577 (48.8) | 1652 (51.2) |  |  | 2953 (44.1) | 3748 (55.9) |  |
| Age (yrs) | 57.08±7.76 | 58.05±7.45 | <0.0001 |  | 53.15±7.81 | 57.27±7.36 | <0.0001 |
| BMI (kg/m2) | 23.35±2.68 | 26.41±2.97 | <0.0001 |  | 22.81±2.96 | 25.86±6.65 | <0.0001 |
| Waist circumference (cm) | 83.40±7.58 | 93.15±10.93 | <0.0001 |  | 77.20±8.81 | 87.31±8.61 | <0.0001 |
| SBP (mmHg) | 128.72±17.95 | 139.34±18.07 | <0.0001 |  | 118.78±18.29 | 135.71±19.33 | <0.0001 |
| DBP (mmHg) | 79.65±10.08 | 85.50±9.90 | <0.0001 |  | 75.29±9.08 | 82.00±10.02 | <0.0001 |
| WHR | 0.88±0.06 | 0.93±0.07 | <0.0001 |  | 0.83±0.09 | 0.89±0.15 | <0.0001 |
| TC | 4.56±0.91 | 4.57±1.07 | 0.592 |  | 4.58±1.03 | 4.79±1.05 | <0.0001 |
| TG | 1.26±0.71 | 2.39±1.76 | <0.0001 |  | 1.10±0.51 | 2.09±1.39 | <0.0001 |
| HDL-c | 1.30±0.33 | 1.06±0.30 | <0.0001 |  | 1.37±0.32 | 1.16±0.26 | <0.0001 |
| LDL-c | 2.60±0.71 | 2.52±0.74 | 0.001 |  | 2.58±0.77 | 2.68±0.80 | <0.0001 |
| FPG | 6.21±1.83 | 6.87±1.91 | <0.0001 |  | 5.62±0.94 | 6.58±1.80 | <0.0001 |
| PPG | 8.20±3.99 | 9.98±4.44 | <0.0001 |  | 7.17±2.54 | 9.65±4.01 | <0.0001 |
| HbA1c | 5.87±1.06 | 6.22±1.17 | <0.0001 |  | 5.73±0.61 | 6.16±1.10 | <0.0001 |
| eGFRb | 135.19±25.71 | 129.45±27.33 | <0.0001 |  | 126.44±24.54 | 118.15±23.41 | <0.0001 |
| MET-h/wk | 21.0 (0-35.6) | 20.0 (0-37.5) | 0.279 |  | 21.0 (0-45.2) | 21.0 (0-39.1) | 0.326 |
| Current smoking, n (%) | 749 (47.5) | 756 (45.8) | 0.341 |  | 37 (1.0) | 36 (1.2) | 0.407 |
| Current alcohol consumption, n (%) | 764 (48.4) | 891 (53.9) | 0.002 |  | 280 (7.5) | 262 (8.9) | 0.038 |
| Postmenopausal women, n (%) |  |  |  |  | 1641 (43.8) | 1035 (35.0) | <0.0001 |

BMI, body mass index; SBP, systolic blood pressure; DBP, diastolic blood pressure; TG, Triglycerides ; TC, total cholesterol; HDL-c, high-density lipoprotein-cholesterol; LDL-c, low-density lipoprotein-cholesterol; FPG, fasting plasma glucose; PPG, postprandial 2-hour plasma glucose; WHR, waist to hip ratio; HbA1c, glycated hemoglobin; eGFR, estimated glomerular filtration rate

^a^Data are means ± SD, median (interquartile range), or number (percent)

^b^This variables was log transformed before analysis.

Supplement Table S1B. Characteristics of Men and Women Stratified by Metabolic Syndrome Status (IDF criteria)

|  | Men (n=3229) | | |  | women (n=6701) | | |
| --- | --- | --- | --- | --- | --- | --- | --- |
|  | MetS (-) | MetS (+) | P value |  | MetS (-) | MetS (+) | P value |
| n (%) | 1592 (49.3) | 1637 (50.7) |  |  | 3190 (47.6) | 3511 (52.4) |  |
| Age (yrs) | 57.11±7.81 | 58.42±7.31 | <0.0001 |  | 53.62±7.77 | 57.38±7.47 | <0.0001 |
| BMI (kg/m2) | 23.49±2.55 | 26.48±2.95 | <0.0001 |  | 22.72±2.94 | 25.53±6.41 | <0.0001 |
| Waist circumference (cm) | 83.89±7.78 | 93.78±10.59 | <0.0001 |  | 78.58±8.52 | 87.92±8.49 | <0.0001 |
| SBP (mmHg) | 128.85±17.69 | 139.21±18.14 | <0.0001 |  | 119.36±18.58 | 135.17±19.42 | <0.0001 |
| DBP (mmHg) | 79.25±10.14 | 85.49±9.97 | <0.0001 |  | 76.17±9.34 | 82.26±10.15 | <0.0001 |
| WHR | 0.87±0.07 | 0.95±0.09 | <0.0001 |  | 0.82±0.10 | 0.91±0.12 | <0.0001 |
| TC | 4.56±0.89 | 4.57±1.05 | 0.603 |  | 4.57±1.05 | 4.75±1.10 | <0.0001 |
| TG | 1.25±0.69 | 2.39±1.68 | <0.0001 |  | 1.11±0.56 | 2.08±1.40 | <0.0001 |
| HDL-c | 1.31±0.32 | 1.05±0.31 | <0.0001 |  | 1.38±0.32 | 1.15±0.28 | <0.0001 |
| LDL-c | 2.61±0.70 | 2.51±0.75 | 0.001 |  | 2.57±0.77 | 2.69±0.81 | <0.0001 |
| FPG | 6.18±1.79 | 6.89±1.93 | <0.0001 |  | 5.60±0.92 | 6.61±1.82 | <0.0001 |
| PPG | 8.17±3.85 | 10.01±4.52 | <0.0001 |  | 7.15±2.51 | 9.69±4.07 | <0.0001 |
| HbA1c | 5.84±1.03 | 6.24±1.18 | <0.0001 |  | 5.71±0.60 | 6.18±1.12 | <0.0001 |
| eGFRb | 135.28±25.69 | 129.12±27.41 | <0.0001 |  | 127.12±24.62 | 117.12±23.09 | <0.0001 |
| MET-h/wk | 21.0 (0-35.5) | 20.0 (0-37.6) | 0.301 |  | 21.0 (0-45.2) | 21.0 (0-39.1) | 0.334 |
| Current smoking, n (%) | 755 (47.4) | 756 (46.2) | 0.481 |  | 37 (1.2) | 36 (1.0) | 0.638 |
| Current alcohol consumption, n (%) | 767 (48.2) | 888 (54.2) | 0.01 |  | 286 (9.0) | 256 (7.3) | 0.014 |
| Postmenopausal women, n (%) |  |  |  |  | 1661 (52.1) | 1015 (28.9) | <0.0001 |

BMI, body mass index; SBP, systolic blood pressure; DBP, diastolic blood pressure; TG, Triglycerides ; TC, total cholesterol; HDL-c, high-density lipoprotein-cholesterol; LDL-c, low-density lipoprotein-cholesterol; FPG, fasting plasma glucose; PPG, postprandial 2-hour plasma glucose; WHR, waist to hip ratio; HbA1c, glycated hemoglobin; eGFR, estimated glomerular filtration rate

a Data are means ± SD, median (interquartile range), or number (percent)

bThis variables was log transformed before analysis.
